# Bintrafusp Alfa, an Anti-PD-L1:TGFβ Trap Fusion Protein, in Patients with ctDNA-positive, Liver-limited Metastatic Colorectal Cancer

**DOI:** 10.1158/2767-9764.CRC-22-0194

**Published:** 2022-09-14

**Authors:** Van K. Morris, Michael J. Overman, Michael Lam, Christine M. Parseghian, Benny Johnson, Arvind Dasari, Kanwal Raghav, Bryan K. Kee, Ryan Huey, Robert A. Wolff, John Paul Shen, June Li, Isabel Zorrilla, Ching-Wei D. Tzeng, Hop S. Tran Cao, Yun Shin Chun, Timothy E. Newhook, Nicolas Vauthey, Dzifa Duose, Raja Luthra, Cara Haymaker, Scott Kopetz

**Affiliations:** 1 Department of Gastrointestinal Medical Oncology, The University of Texas MD Anderson Cancer Center, Houston, Texas.; 2 Department of Medical Oncology and Cancer Services, The University of Melbourne, Melbourne, Australia.; 3 Department of Translational Molecular Pathology, The University of Texas MD Anderson Cancer Center, Houston, Texas.; 4 Department of Surgical Oncology, The University of Texas MD Anderson Cancer Center, Houston, Texas.; 5 Department of Pathology and Laboratory Medicine, The University of Texas MD Anderson Cancer Center, Houston, Texas.

## Abstract

Identification of circulating tumor DNA (ctDNA) following curative intent therapies is a surrogate for microscopic residual disease for patients with metastatic colorectal cancer (mCRC). Preclinically, in micrometastatic microsatellite stable (MSS) colorectal cancer, increased TGFβ signaling results in exclusion of antitumor cytotoxic T cells from the tumor microenvironment. Bintrafusp alfa (BA) is a bifunctional fusion protein composed of the extracellular domain of the TGFβRII receptor (“TGFβ trap”) and anti-PD-L1 antibody. Patients with liver-limited, MSS mCRC and with detected ctDNA after complete resection of all known tumors and standard-of-care therapy were treated with 1,200 mg of BA intravenously every 14 days for six doses. The primary endpoint was ctDNA clearance. Radiographic characteristics at recurrence were compared using independent *t* tests to historical data from a similar cohort of patients with liver-limited mCRC who underwent observation. Only 4 of 15 planned patients received BA before the study was stopped early for loss of equipoise. There was no grade ≥3 adverse event. None of the patients cleared ctDNA. All patients developed radiographic recurrence by the first planned restaging. Although not detectable at prior to treatment, TGFβ3 was found in circulation in all patients at cycle 2 day 1. Compared with a historical cohort, patients administered BA developed more metastases (15 vs. 2, *P* = 0.005) and greater tumor volumes (9 cm vs. 2 cm, *P* = 0.05). Treatment with BA in patients with ctDNA-detected, liver-limited mCRC did not clear ctDNA and was associated with large-volume recurrence, highlighting the potential context-specific complexity of dual TGFβ and PD-L1 inhibition.

**Significance:**

Use of ctDNA to identify patients with micrometastatic disease for therapeutic intervention is feasible. Treatment with BA in patients with liver-limited mCRC and with detectable ctDNA after resection generated rapid progression. Approaches targeting TGFβ signaling must consider its pathway complexity in future immunotherapy combination strategies.

## Introduction

Colorectal cancer remains the second leading cause of cancer-related mortality in the United States, with approximately 50,000 deaths expected in 2021 (1). Most patients with metastatic colorectal cancer (mCRC) will develop liver metastases, which account for almost two-thirds of all colorectal cancer deaths (2). While increased use of hepatic resection (3–5) and the advent of biologic agents targeting VEGF and EGFR have improved long-term survival outcomes for the 15% to 25% of patients with mCRC who have resectable liver-limited disease, 5-year survival rates for these patients still range between 20% and 40% (6). Approximately 75% of patients with mCRC who undergo resection of liver metastases will develop disease recurrence (7). Novel approaches are therefore needed so that patients who are at high risk for recurrence can be identified earlier and offered more effective therapeutics.

Circulating tumor DNA (ctDNA) is released by tumor cells into the circulation predominantly via apoptosis (8). With a half-life on the order of hours (9), ctDNA can reveal somatic mutations harbored by tumor cells that are not present in nonmalignant cells and can serve as a “real-time” indicator of persistent cancer. The ability to detect a variant allele fraction (VAF) in the ctDNA of as low as 0.1% equips clinicians with a highly sensitive approach for identifying the presence of any microscopic foci of tumor cells (10). Indeed, in patients with colorectal cancer who undergo complete surgical resection, the detection of ctDNA is associated with inevitable disease recurrence and therefore serves as a surrogate for the existence of persistent minimal residual (micrometastatic) disease (11–15).

For the more than 95% of patients with unresectable mCRC characterized by microsatellite stability (16), immune checkpoint inhibitors targeting programmed death-1 (PD-1) and programmed death-ligand 1 (PD-L1) are ineffective, with reported overall response rates of less than 5% (17, 18). One reason for this lack of response to immunotherapy for microsatellite-stable mCRC is the absence of cytotoxic immune cells within the tumor microenvironment, which renders these tumors immunologically unreactive (19–23). *In vivo*, preclinical syngeneic models of colorectal cancer have demonstrated that micrometastatic tumor deposits feature activated CD4^+^ and CD8^+^ T cells that are not present in macroscopic tumors (24). In these models, this “immune exclusion” in the tumor microenvironment is mediated by upregulating signaling of the TGFβ pathway, and dual targeting of PD-L1 and TGFβ increased CD4^+^ and CD8^+^ T-cell infiltrates within the tumor microenvironment, which was not seen with inhibition of single targets alone (25).

On this basis, we hypothesized that prevention of TGFβ-induced immune exclusion along with immune checkpoint blockade may eliminate colorectal cancer micrometastases still present after resection of all evident disease. Bintrafusp alfa (BA), a bifunctional fusion protein composed of the extracellular domain of the TGFβ receptor II fused to a human IgG1 antibody blocking PD-L1, has demonstrated clinical activity and has a manageable safety profile in patients with solid tumors (26, 27). Using “ctDNA-positive” status as a surrogate for remnant micrometastatic colorectal cancer, we conducted a pilot study in patients with liver-limited mCRC who had no clinically evident disease following resection and completion of all standard-of-care therapy, to determine whether BA treatment led to clearance of ctDNA (and presumably, of micrometastatic disease).

## Materials and Methods

### Study Design and Participants

This trial was a prospective, single-arm pilot study of BA as monotherapy conducted under Institutional Review Board approval at The University of Texas MD Anderson Cancer Center (Houston, TX). All participants signed written informed consent prior to any study-related treatment or procedure. Patients over the age of 18 years with liver-limited metastatic adenocarcinoma of the colon or rectum who had undergone an R0 (complete) resection of their primary tumor and all known liver metastases and who had received all planned standard-of-care perioperative therapy (e.g., chemotherapy and/or radiotherapy), at the discretion of the multidisciplinary team of providers, were tested for ctDNA status (i.e., “ctDNA detected” or “not detected”). ctDNA from isolated plasma was analyzed using a 70-gene capture-based, next-generation sequencing panel of total size 150 kb (Supplementary Fig. S1) according to methodology described previously (28) and approved for use in a Clinical Laboratory Improvement Amendment (CLIA) environment. Using molecular barcoding, this panel is able to detect single-nucleotide variants in all 70 genes, copy-number variations in 19 genes, insertion-deletions in 22 genes, and fusions in six genes. ctDNA was tested at least 14 days after completion of all standard-of-care therapy. Only patients with at least one mutation detectable in the ctDNA were eligible. Only patients whose colorectal cancer had been characterized as microsatellite stable on the basis of IHC analysis showing expression of MLH1, MSH2, MSH6, and PMS2 were eligible. Patients were also required to have an Eastern Cooperative Oncology Group performance status of 0 or 1 and an estimated life expectancy exceeding 12 weeks according to the judgment of the investigator. Patients must not have had any radiographic evidence of disease at the time of study entry according to CT or MRI.

In addition, included patients must have had adequate hematologic function for study participation, defined as an absolute neutrophil count ≥1.0 × 10^9^/L, absolute lymphocyte count ≥0.5 × 10^9^/L, platelet count ≥100 × 10^9^/L, and hemoglobin level ≥9.0 g/dL. In addition, patients must have had adequate renal function (defined as an estimated creatinine clearance > 30 mL/minute according to the Cockcroft-Gault formula) and adequate hepatic function [defined as a total bilirubin level ≤1.5 × the upper limit of normal (ULN), an aspartate aminotransferase level ≤2.5 × ULN, and an alanine aminotransferase level ≤2.5 × ULN].

Patients were ineligible if they had a history of extrahepatic metastases of colorectal cancer. Patients could not have had prior exposure to an immune checkpoint inhibitor or any other antineoplastic immunomodulatory agent. Patients with a history of a second primary malignancy within 3 years of study treatment were ineligible, as were patients who had undergone prior organ transplantation that necessitated ongoing immunosuppression. Patients with an active infection—including human immunodeficiency virus, hepatis B virus, hepatitis C virus, and tuberculosis—were ineligible. In addition, patients with active autoimmune disease with the potential for clinical deterioration upon treatment with BA, at the discretion of the evaluating investigator, were not allowed to participate in the study. Pregnant women were not eligible. All research conducted as a part of this clinical trial was performed in accordance with the Declaration of Helsinki, and this trial is registered at ClinicalTrials.gov (NCT03436563).

### Procedures

**FIGURE 1:**
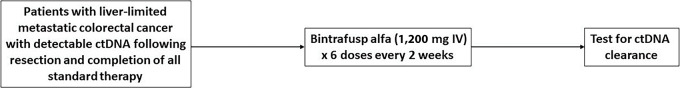
Study schema.

BA (EMD Serono) was administered intravenously every 14 days at a fixed dose of 1,200 mg (Fig. 1). Toxicity was evaluated at baseline and prior to each administration of BA. Dose reductions were not permitted. Adverse events were evaluated using the NCI Common Terminology Criteria for Adverse Events (CTCAE), version 4.03 (29). Treatment with BA continued for a total of six planned doses, or until one of the following events (whichever came first): therapeutic failure requiring urgent additional antineoplastic therapy, unacceptable toxicity, onset of pregnancy, or withdrawal of informed consent.

Two weeks after completion of therapy with BA, patients were evaluated for treatment response with repeat ctDNA analysis. Patients also underwent radiographic restaging studies at this time, and every 3 months thereafter, to detect any disease recurrence.

### Outcomes

The primary endpoint for this study was clearance of ctDNA, defined as the disappearance of all somatic mutations identified in the blood, as well as no appearance of any new somatic mutations, following six doses of BA. Secondary endpoints were disease-free survival (DFS), calculated as the time from the date of first administration of BA until the date of documented recurrence or development of distant metastasis by RECIST (30); overall survival (OS), calculated as the time from the date of first administration of BA to the date of death by any cause; and the occurrence of grade 3 or higher adverse events according to CTCAE version 4.03.

### Statistical Analysis

The planned sample size for this pilot study was 15 patients. Descriptive statistics were used to estimate the proportion of patients with ctDNA clearance, along with the associated 95% confidence interval (CI). Median DFS and OS durations (with associated 95% CIs) were estimated according to the Kaplan–Meier method (GraphPad software, version 8 was used for statistical analyses.

### Circulating Biomarker Analysis

TGFβ1, 2, and 3 expression were measured in patients’ plasma, which was separated from whole blood. The other 40 soluble proteins (Supplementary Table S1) were measured in serum. Frozen plasma or serum aliquots stored in −80°C were thawed on ice immediately before performing the assay. These biomarkers were measured on the basis of multiplex electrochemiluminescence detection assays using commercially available kits from Meso Scale Discovery. The U-PLEX TGFβ Combo Human kit (K15241K-1) and V-PLEX Human Biomarker 40-Plex Kit (K15209D-1) were used. The assays were performed following the manufacturer's instructions.

Sample acquisition was performed using a QuickPlex SQ 120 instrument and analyzed using the DISCOVERY WORKBENCH 4.0 software. All samples from the same patient were run on the same plate and samples were run in technical triplicates. The results were graphed using Prism 8.0 software.

### Analysis of Standard-of-care Cohort

In an unplanned, *post hoc* analysis, under an Institutional Review Board–approved protocol, we retrospectively reviewed databases at MD Anderson Cancer Center for patients with liver-limited mCRC in whom ctDNA was detected before surgical resection and who proceeded to observation following complete resection and standard-of-care chemotherapy. Data from these patients’ electronic medical records were obtained, including demographics, DFS, OS, vital status, and characteristics of tumor recurrence (including the number and sizes of metastases at the time of recurrence). To maintain consistency for comparison of tumor volumes across both cohorts, size dimensions of metastases were measured using the same principles for measuring target lesions according to RECIST 1.1 (31) for the purposes of estimating tumor volume. The mean number of metastases at recurrence and mean total tumor burden, defined by the sum of measurable target lesions, were compared with those for patients treated with BA using an independent *t* test. IBM SPSS Statistics software, version 26 was used for these analyses. Differences with a *P* value <0.05 were considered statistically significant.

### Data Availability

All data generated in this study are available within this article and Supplementary Data.

## Results

Four patients received BA on this study. As shown in Supplementary Table S2, their median age was 55.9 years (range, 42.5–68.7). Two patients had right-sided primary colon cancers, and 2 patients had sigmoid colon cancers. The median number of liver metastases at initial presentation was 4 (range, 2–4). Two patients’ tumors had *KRAS^G12D^* mutations, whereas the remaining 2 patients harbored colorectal cancers expressing wild-type *KRAS*. All primary tumors expressed wild-type *NRAS* and *BRAF*. The median carcinoembryonic antigen (CEA) level prior to BA initiation was 4.6 ng/mL.

The median number of doses of BA received was 6 (range, 3–6). Overall, BA was tolerated well, with no grade 3 or higher treatment-related adverse events observed (Table 1). The most common adverse event was dermatitis (*n* = 3; all grade 1). One patient developed a keratoacanthoma of the skin, and another developed a squamous cell carcinoma of the skin. Both of these are likely related to the TGFβ trap component of the BA agent, given known linkage between disruption of TGFβ homeostasis and hyperproliferation of skin squamous epithelium (32).

**TABLE 1:** Adverse events according to CTCAE, version 4.03*

	Grade 1 (No.)	Grade 2 (No.)
Rash	3	0
Squamous cell carcinoma of skin	0	1
Actinic keratosis	1	0
Anorexia	1	0
Arthralgia	1	0
Condyloma	1	0
Congestion-nasal	1	0
Creatine kinase, increased	1	0
Diarrhea	1	0
Epistaxis	1	0
Fatigue	1	0
Flu-like symptoms	1	0
Hypothyroidism	1	0
Mucositis	1	0
Myalgia	1	0

**Abbreviations:** BA, bintrafusp alfa; CEA, carcinoembryonic antigen; CI, confidence interval; CLIA, Clinical Laboratory Improvement Amendment; CRC, colorectal cancer; CTCAE, Common Terminology Criteria for Adverse Events; ctDNA, circulating tumor DNA; DFS, disease-free survival; EGFR, epidermal growth factor receptor; mCRC, metastatic colorectal cancer; OS, overall survival; PD-1, programmed death-1; PD-L1, programmed death ligand-1; TGF, transforming growth factor; ULN, upper limit of normal; VAF, variant allele fraction; VEGF, vascular endothelial growth factor.

*No grade 3 or 4 adverse events occurred.

**FIGURE 2:**
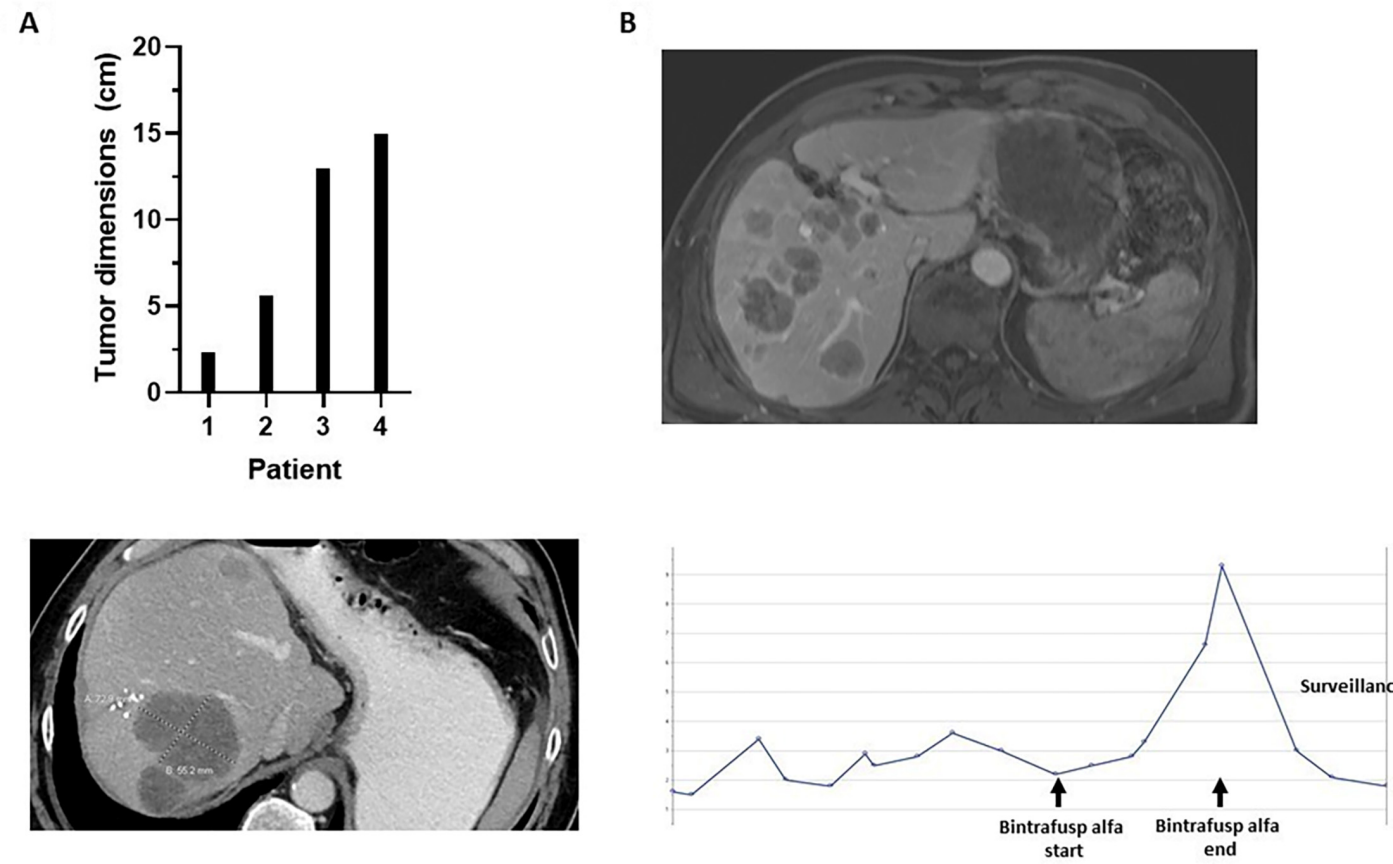
Tumor characteristics upon recurrence after BA treatment. **A,** Tumor sizes. **B** and **C,** Images by CT of hepatic metastases in two different patients. **D,** Trend in carcinoembryonic antigen levels in one patient before, during, and after BA treatment.

At the time of first restaging following completion of study treatment, all patients had radiographic evidence of disease recurrence (Fig. 2A). The first patient developed a single new liver metastasis measuring approximately 1 cm in its greatest diameter. The second patient developed multifocal new metastases throughout the liver, the largest measuring 7 cm in its greatest diameter. This patient also developed treatment-unrelated diverticulitis within 2 months of study discontinuation that was complicated by an extended recovery. During this time, he did not receive antineoplastic therapy for his mCRC. While off chemotherapy, his CEA level dropped from a level of 9.3 ng/mL back to within normal levels (1.5 ng/mL), and serial imaging studies showed no further growth in the size of his tumors. The third patient (Fig. 2B) developed more than 30 new liver metastases, 10 new lung metastases, and distant lymph node metastases following 3 months of treatment with BA. The fourth patient developed four new liver metastases after three doses of BA (Fig. 2C), with restaging studies conducted early at 6 weeks due to clinical suspicion of recurrence based upon a rising CEA level (Fig. 2D).

The rate of ctDNA clearance following treatment with BA was 0% (95% CI, 0–60). Table 2 details the changes in ctDNA mutation profiles from baseline (before BA treatment initiation) to disease recurrence. The median number of tumor-specific mutations in the ctDNA at study enrollment was 2 (range, 1–5), with a maximum VAF of 0.5% (TP53^R1^^96^^*^ and *APC^A703fs^*) for any of the mutations detected in the pretreatment ctDNA. Three patients had plasma available for ctDNA analysis at the time of radiographic disease recurrence. In each of these patients, mutations detected before treatment were retained at recurrence, albeit often at a much higher VAF. For example, for patient 2, VAF increases were observed for APC^R876*^ (<0.3% to 64.7%), *KRAS^G12D^* (<0.3% to 60.8%), and TP53^R196*^ (0.5% to 65.7%). Several new mutations were also observed at the time of radiographic disease recurrence. Patient 3, who only had a *TP53^C238Y^* mutation (VAF 0.3% at baseline) developed mutations in APC^R1450^^*^ (VAF 23.3%), APC^R216*^ (VAF 23.0%), *KRAS^G12D^* (VAF 21.0%), *SMAD4^D335G^* (VAF 31.5%), *MAPK1^Q97K^* (VAF 1.2%), *STK11^D330E^* (VAF 0.4%), and *KIT^R804W^* (VAF <0.2%) after six doses of BA.

**TABLE 2:** Changes in ctDNA mutation profiles (with associated variant allele fractions) before treatment with BA and following recurrence

Patient	Mutation	Pretreatment VAF (%)	Postrecurrence VAF (%)
1	*APC^A703fs^*	0.5	0.4
	*TP53^P278fs^*	<0.3	0.3
	*TP53^C277G^*	0.4	—
2	TP53^R196*^	0.5	65.7
	APC^R876*^	<0.3	64.7
	*KRAS^G12D^*	<0.3	60.8
	*MET^N786fs^*	0.3	<0.3
	*MTOR^R206H^*	<0.3	—
	*BRCA2^D1360Y^*	—	0.3
3	*TP53^C238Y^*	0.3	<0.2
	*SMAD4^D335G^*	—	31.5
	APC^R1450*^	—	23.3
	APC^R216*^	—	23.0
	*KRAS^G12D^*	—	21.0
	*MAPK1^Q97K^*	—	1.2
	*STK11^D330E^*	—	0.4
	*KIT^R804W^*	—	<0.2
4	*ERBB2^R288W^*	<0.2	(not tested)

Abbreviation: VAF, variant allele fraction.

**FIGURE 3:**
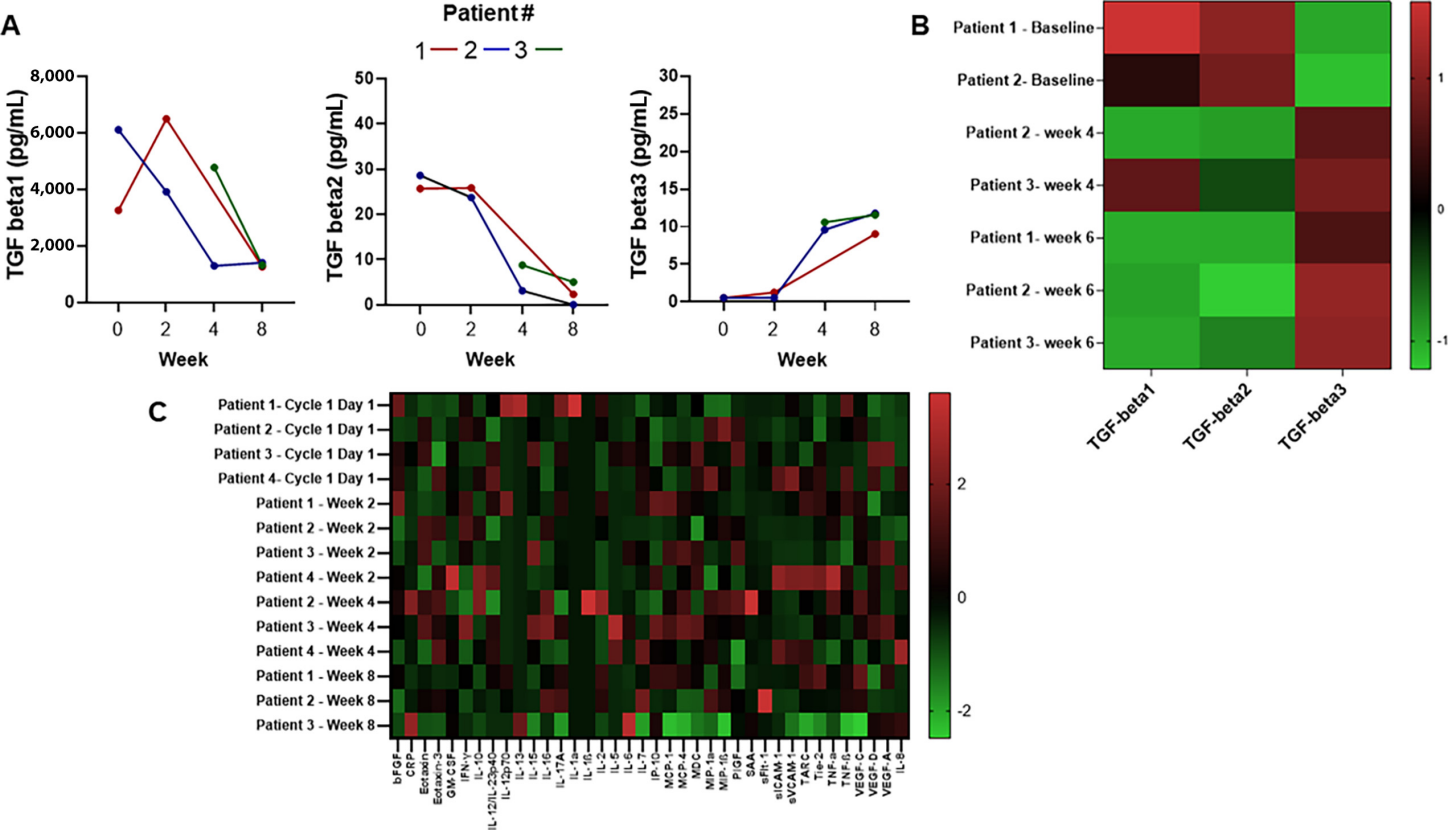
Cytokine and chemokine changes over time: TGFβ 1, 2, and 3 concentrations in plasma collected over time for individual patients (**A**); heatmap of normalized TGFβ 1, 2, and 3 concentrations in plasma at serial timepoints for individual participants (**B**); and heatmap of additional cytokines and chemokines assessed at serial timepoints in patient serum (**C**).

We assessed changes in cytokine and chemokines in circulation, including TGFβ isoforms. Cytokine and chemokine assessment in all patients revealed an emergence of detectable TGFβ3 in patient plasma by cycle 2, day 1 (Fig. 3A and B). However, TGFβ1 and TGFβ2 detection was reduced as expected according to the mechanism of drug action. Of the additional 40 cytokines and chemokines assessed in patient serum over time, no pattern or significant change was elsewhere observed (Fig. 3C).

**FIGURE 4:**
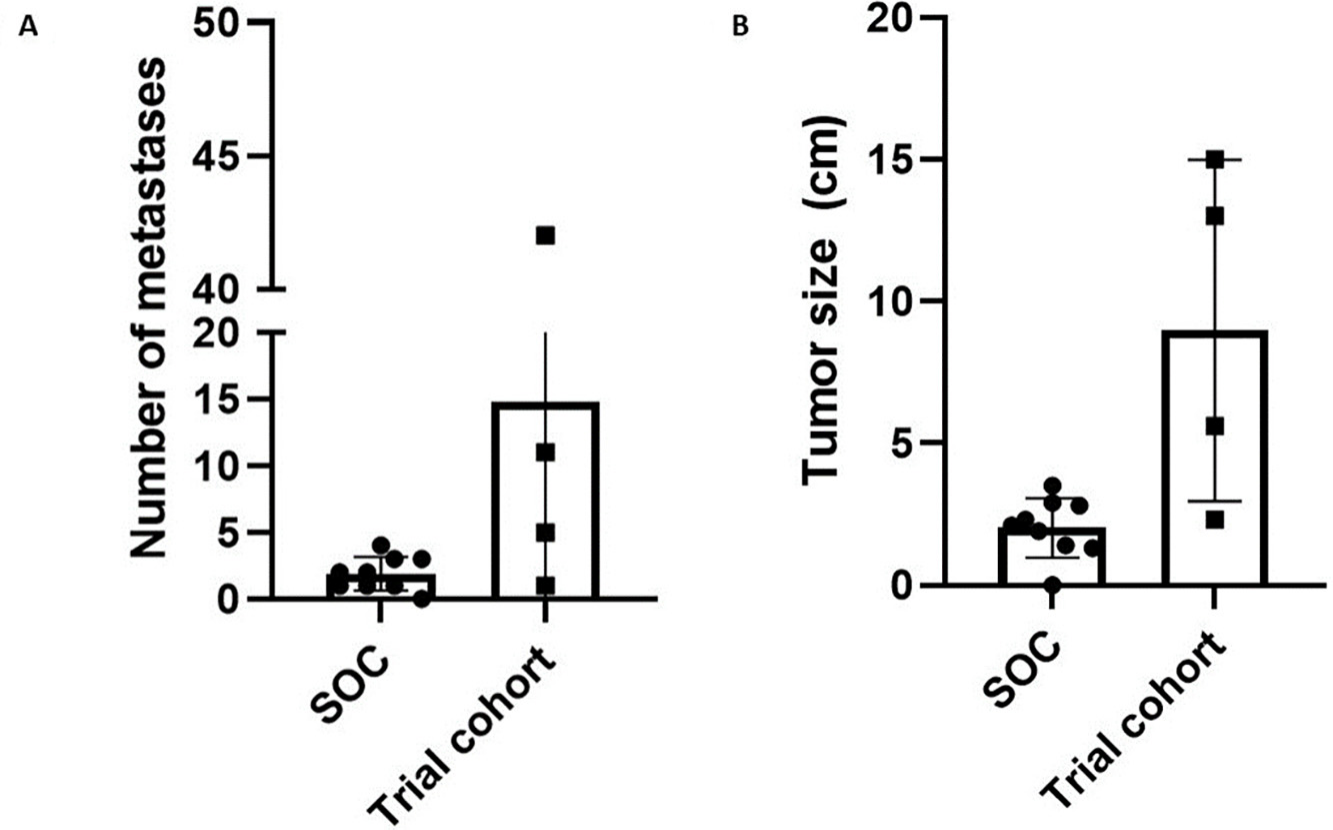
Measurements of number of metastases (**A**) and tumor volume (**B**) for patients with liver-limited metastatic colorectal cancer who received standard-of-care (SOC) observation or BA (trial cohort).

On the basis of the concerns about the rapidity and aggressiveness of the recurrences in these patients, the study team reviewed the historical recurrence patterns for patients with detectable ctDNA in patients who had previously undergone resection for liver-limited mCRC. We retrospectively identified 9 patients who had detectable ctDNA (using the same assay used for the prospective study) following completion of all standard-of-care therapy (Supplementary Table S3). There was no difference in number of liver metastases at the time of initial presentation (prior to surgery), mean size of metastases at initial presentation, age, primary tumor sidedness, or KRAS/NRAS/BRAF mutation status to suggest baseline differences in the two populations of patients (Supplementary Table S4). The mean time to the first restaging scan after completion of all planned therapies/start of observation was 3.0 months and did not differ from that of patients treated with BA (*P* = 0.83). While on observation, radiographic recurrence was detected in 8 of these patients. The median DFS for these patients was 4.2 months (Supplementary Fig. S2), which was somewhat longer than the median DFS of 3.0 months for patients treated with BA (HR: 4.9; 95% CI: 0.9–27.0; *P* = 0.07). At the time of recurrence, patients with ctDNA(+) liver-limited mCRC who proceeded to observation had a lower mean number of total metastatic lesions (2 vs. 15, *P* = 0.05; Fig. 4A) and smaller mean total tumor burden (2.0 cm vs. 9.0 cm, *P* = 0.005; Fig. 4B) than did those who received BA. Because of investigator concern that BA may be clinically detrimental, the decision was made to close the study to new patient accrual after treatment of 4 patients.

## Discussion

Here we report the clinical results from the first study, to our knowledge, to target micrometastatic colorectal cancer, informed by detection of ctDNA as a surrogate for minimal residual disease, by concomitant targeting of TGFβ signaling and PD-L1 blockade in patients with liver-limited mCRC. While BA was overall well tolerated, the rapid onset of clinical progression and the acquisition of new mutations raised concern among investigators for loss of equipoise, leading to premature discontinuation of the study.

Identification of ctDNA in the plasma following complete resection and subsequent adjuvant therapies has been demonstrated to be a biomarker of poor prognosis—a harbinger of inevitable recurrence—for patients with all stages of colorectal cancer and those with several other solid tumors (11–15, 33, 34). In one series of patients with liver-limited mCRC who underwent surgical resection of all evident disease, patients in whom ctDNA was detected in the postoperative setting had a significantly lower 2-year recurrence-free survival rate than did patients with no detectable ctDNA (0% vs. 47%, respectively; ref. 15). Consistent with this trend, our retrospective cohort of patients with liver-limited mCRC developed recurrence in most (89%) cases. Despite the strong prognostic implications of detection of ctDNA prior to eventual clinical recurrence, the clinical utility of this methodology as a predictive biomarker for response to further treatment has yet to be demonstrated, owing to a lack, thus far, of reported prospective intervention trials.

In our study, which sought to use a novel combinatory immunotherapy approach to eradicate minimal residual disease as indicated by ctDNA, the rapid onset of disease recurrence in patients with liver-limited mCRC was unexpected. Here, the 4 patients treated with BA developed higher tumor burdens, both in terms of total disease volume and the number of metastatic lesions, than was observed in a similar cohort of patients who proceeded to surveillance with no BA treatment. The occurrence of hyperprogression with immune checkpoint inhibitors has been reported in several series across solid tumors, with a prevalence of between 5% and 20% (35–37). It is possible that rapid tumor growth noted in our study was attributable to the use of anti-PD-L1 therapy and by removal of circulating TGFβ1 and TGFβ2 with the TGFβ trap of BA. However, to our surprise, all patients assessed showed an increased detection of TGFβ3 in circulation by cycle 2 day 1, a trend opposite that observed for TGFβ1 and TGFβ2. Despite significant homology in its primary structure with the other TGFβ isoforms (38), TGFβ3 is distinguished by a more “open” interaction with the TGFβ receptor II that may generate TGFβ3-specific oncogenic activation (39). While not fully characterized in colorectal cancer, inhibition of TGFβ3 in preclinical models of glioblastoma multiforme is associated with decreased expression of downstream SMAD oncogenes, decreased tumor invasiveness, and dampened TGFβ1/TGFβ2 signaling (40). The rise of circulating TGFβ3 observed in all patients following treatment with BA in our study raises concern for the possibility of a compensatory escape mechanism linked to the rapid clinical progression observed.

Interestingly, the rapid tumor growth in one patient that had been observed while on treatment with BA appeared to plateau after study drug discontinuation. The patient, whose tumor harbored a *KRAS^G12D^* mutation, was monitored off systemic antineoplastic therapy for several months with minimal change, a pattern of disease biology that is not consistent with his aggressive recurrence. Murine models of *KRAS^G12D^* colorectal cancer have shown that upregulation of signaling in the MAPK pathway with oncogenic *KRAS* mutations promotes tumor dedifferentiation for which TGFβ signaling may compensate against tumorigenesis (41). Furthermore, subsequent blocking of the type I TGFβ receptor disrupted this compensatory feedback and greatly accelerated tumor growth. This preclinical observation suggests that, *in vivo*, activation of MAPK signaling with impairment of prodifferentiation TGFβ activity may promote dedifferentiation and rapid development of colorectal tumors. While we were unable to compare TGFβ3 expression in the tumor tissue with that found in circulation, our observations collectively are consistent with our findings here that the treatment of micrometastatic, liver-limited mCRC with BA may have promoted tumor hyperprogression for the patients on this study.

Furthermore, TGFβ has been implicated *in vivo* to promote exclusion of CD4^+^ and CD8^+^ T cells within the tumor microenvironment (24). Upregulation of TGFβ signaling concurrent with growth of colorectal metastases favors creation of an immunologically inactive tumor milieu that is unresponsive to immune checkpoint blockade. Our hypothesis was that removal of TGFβ using this TGFβ trap would delay TGFβ-mediated immune suppression and render micrometastases susceptible to immune-mediated cytotoxicity driven by blockade of the PD-1–PD-L1 interaction. Unfortunately, targeting TGFβ signaling with BA in patients with micrometastatic colorectal cancer did not clear the ctDNA, nor did it promote sustained disease-free survival for these patients. *In vivo*, unaffected liver parenchyma harbors monocyte-derived macrophages which bind and trigger apoptosis of CD8^+^ T cells upon a Fas–Fas ligand interaction in our study (42). Therefore, it is possible that selection of patients in our trial specifically with liver-limited mCRC at high risk for recurrence within the liver may have been predisposed to increased hepatic clearance of T cells from the circulation that made eradication of the microscopic tumor deposits more difficult.

Our study demonstrates the feasibility of designing clinical trials seeking to eliminate micrometastatic disease, represented by ctDNA status, for patients with colorectal cancer. However, the small sample size of study participants (*N* = 4) limits the generalizability in drawing definitive conclusions toward concomitant targeting of the PD-1–PD-L1 axis and TGFβ signaling in the treatment of microscopic colorectal cancer. We also recognize the limitation of the small cohorts of patients represented here in making definitive comparisons of the radiographic and biochemical changes that were noted upon treatment of micrometastatic colorectal cancer with BA. The rapid increase in tumor burden upon dual targeting of PD-L1 and TGFβ and the accompanying rise in circulating TGFβ3 generate hypotheses for future study of treatment strategies of colorectal cancer which may be better informed by the observed complexity of potential compensatory signaling of multiple TGFβ isoforms (43–45).

## Supplementary Material

Table S1 Supplemental Table S1

Table S2 Supplemental Table S2

Table S3 Supplemental Table S3

Table S4 Supplemental Table S4

Figure SF1 Supplemental Figure S1

Figure SF2 Supplemental Figure S2
