# Supplementary material for: Bintrafusp Alfa, an Anti-PD-L1:TGFβ Trap Fusion Protein, in Patients with ctDNA-positive, Liver-limited Metastatic Colorectal Cancer
Source: Cancer Res Commun. 2022 Sep 14;2(9):979–86. doi: 10.1158/2767-9764.CRC-22-0194 (PMC9648419; doi:10.1158/2767-9764.CRC-22-0194)
Supplement: Table S1 — Supplemental Table S1 [file crc-22-0194-s01.docx]

| **Panel name** | **Biomarkers constituting the panel** |
| --- | --- |
| Angiogenesis Panel (human) | VEGF-A, VEGF-C, VEGF-D, Tie-2, Flt-1, PlGF, and FGF. |
| Chemokine Panel (human) | Eotaxin, MIP-1β, Eotaxin-3, TARC, MIP-1α, MCP-1, MDC, MCP-4, IP-10 and IL-8 |
| Cytokine Panel (human) | GM-CSF, IL-1α, IL-5, IL-7, IL-12/IL-23p40, IL-15, IL-16, IL-17A, TNF-β, VEGF-A |
| Proinflammatory Panel (human) | IFN-γ, IL-1β, IL-2, IL-4, IL-6, IL-8, IL-10, IL-12p70, IL-13, TNF-α |
| Vascular Injury Panel (human) | SAA, CRP, VCAM-1, ICAM-1 |
