# Supplementary material for: Bintrafusp Alfa, an Anti-PD-L1:TGFβ Trap Fusion Protein, in Patients with ctDNA-positive, Liver-limited Metastatic Colorectal Cancer
Source: Cancer Res Commun. 2022 Sep 14;2(9):979–86. doi: 10.1158/2767-9764.CRC-22-0194 (PMC9648419; doi:10.1158/2767-9764.CRC-22-0194)
Supplement: Table S2 — Supplemental Table S2 [file crc-22-0194-s02.docx]

|  | **Patient number** | | | |
| --- | --- | --- | --- | --- |
|  | **1** | **2** | **3** | **4** |
| Age (years) | 42.5 | 68.7 | 66.9 | 44.9 |
| Sex | Male | Male | Male | Male |
| Timing of metastases | Synchronous | Synchronous | Synchronous | Synchronous |
| Primary colon tumor location | Sigmoid colon | Cecum | Cecum | Sigmoid colon |
| *KRAS* mutation status | Wild type | G12D | G12D | Wild type |
| *NRAS* mutation status | Wild type | Wild type | Wild type | Wild type |
| *BRAF* mutation status | Wild type | Wild type | Wild type | Wild type |
| Microsatellite status | Stable | Stable | Stable | Stable |
| Number of initial liver metastases | 2 | 4 | 4 | 4 |
| Diameter of initial largest liver metastasis (cm) | 2.4 | 2.6 | 3.5 | 13 |
| Perioperative systemic chemotherapy | FOLFOX/  bevacizumab | FOLFOX, FOLFIRI | FOLFOX/ bevacizumab | FOLFIRI/  cetuximab, FOLFOX/ bevacizumab |
| Primary tumor T category (ypTx) | T3 | T4 | T3 | T4 |
| Primary tumor N category (ypNx) | N2 | N2 | N2 | N0 |
| No. of lymph nodes with tumor/examined | 10/53 | 5/25 | 5/61 | 0/49 |
| Colorectal tumor differentiation | Poor | Moderate | Moderate | Moderate |
| Postoperative CEA (ng/mL) | 5.6 | 2.4 | 3.6 | 20.1 |
| Doses of bintrafusp alfa | 6 | 6 | 6 | 3 |

Abbreviations: FOLFOX, leucovorin, 5-fluorouracil, and oxaliplatin; FOLFIRI, leucovorin, 5-fluorouracil, and irinotecan; CEA, carcinoembryonic antigen
