# Supplementary material for: Bintrafusp Alfa, an Anti-PD-L1:TGFβ Trap Fusion Protein, in Patients with ctDNA-positive, Liver-limited Metastatic Colorectal Cancer
Source: Cancer Res Commun. 2022 Sep 14;2(9):979–86. doi: 10.1158/2767-9764.CRC-22-0194 (PMC9648419; doi:10.1158/2767-9764.CRC-22-0194)
Supplement: Table S3 — Supplemental Table S3 [file crc-22-0194-s03.docx]

| **Standard-of-care (observation)** | | | | | | | | | | **Bintrafusp alfa** | | | | |
| --- | --- | --- | --- | --- | --- | --- | --- | --- | --- | --- | --- | --- | --- | --- |
| **Patient** | **1** | **2** | **3** | **4** | **5** | **6** | **7** | **8** | **9** | **1** | **2** | **3** | **4** |  |
| Recur? | Yes | Yes | Yes | Yes | Yes | Yes | Yes | Yes | No | Yes | Yes | Yes | Yes |  |
| Tumor size by RECIST (cm) | 2.1 | 1.4 | 1.9 | 2.9 | 3.5 | 2.3 | 1.3 | 2.8 | 0 | 2.3 | 13 | 15 | 5.6 |  |
| Organs involved at recurrence | Liver | Liver | Lung | Liver | Liver | Liver | Lymph nodes | Peritoneum | -- | Liver | Liver, lung, lymph nodes | Liver, lymph nodes | Liver, lung |  |
