# Supplementary material for: Bintrafusp Alfa, an Anti-PD-L1:TGFβ Trap Fusion Protein, in Patients with ctDNA-positive, Liver-limited Metastatic Colorectal Cancer
Source: Cancer Res Commun. 2022 Sep 14;2(9):979–86. doi: 10.1158/2767-9764.CRC-22-0194 (PMC9648419; doi:10.1158/2767-9764.CRC-22-0194)
Supplement: Table S4 — Supplemental Table S4 [file crc-22-0194-s04.docx]

|  | **Standard-of-care/ observation**  **(N=9)** | **Bintrafusp alfa**  **(N=4)** | **P-value** |
| --- | --- | --- | --- |
|  |  |  |  |
| Number of liver metastases (mean, SD) | 2.2 (1.4) | 3.3 (0.96) | 0.21 |
| Size of liver metastases (mean, SD) | 4.4 (2.5) | 7.8 (7.6) | 0.23 |
| Age (years) at initial detection of liver metastases (mean, SD) | 62 (10) | 55 (14) | 0.30 |
| Left-sided primary tumor (%) | 22 | 50 | 0.16 |
| *KRAS*/*NRAS*/*BRAF*^wild-type^ status (%) | 56 | 50 | 0.43 |
