# Supplementary figures and images for: Bintrafusp Alfa, an Anti-PD-L1:TGFβ Trap Fusion Protein, in Patients with ctDNA-positive, Liver-limited Metastatic Colorectal Cancer
Source: Cancer Res Commun. 2022 Sep 14;2(9):979–86. doi: 10.1158/2767-9764.CRC-22-0194 (PMC9648419; doi:10.1158/2767-9764.CRC-22-0194)

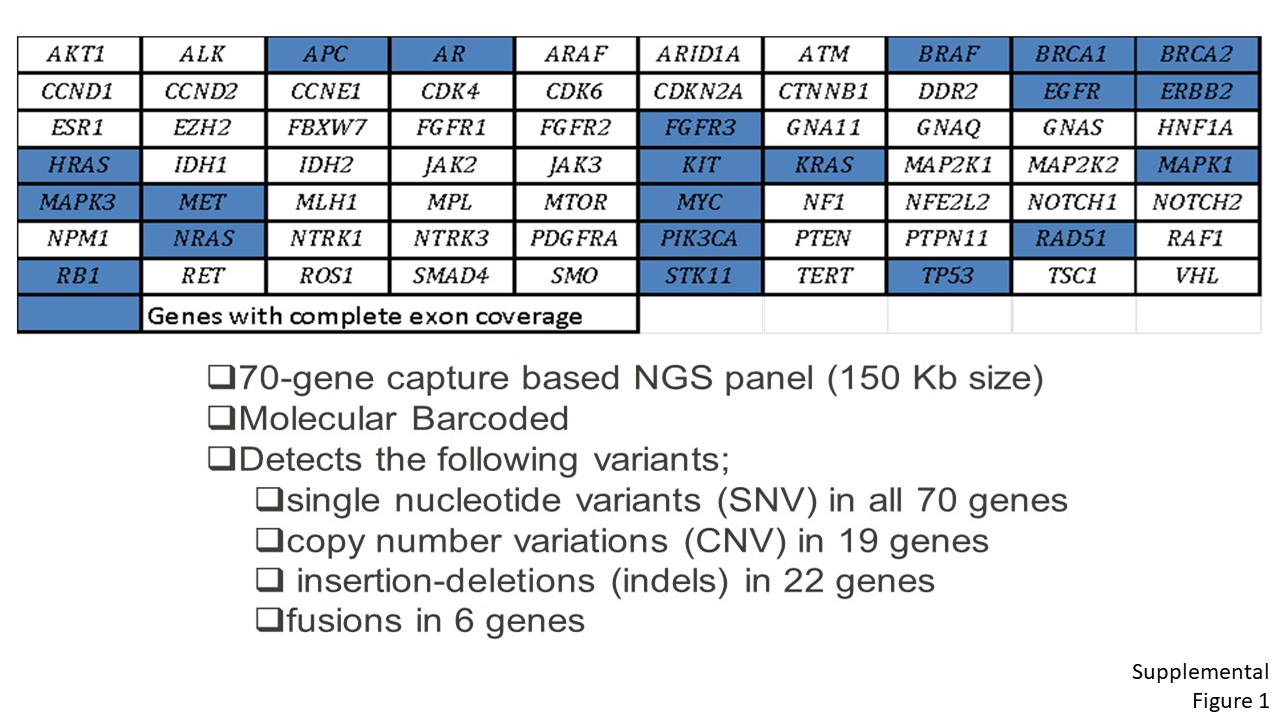

Supplement: Figure SF1 — Supplemental Figure S1 [file crc-22-0194-s05.png]

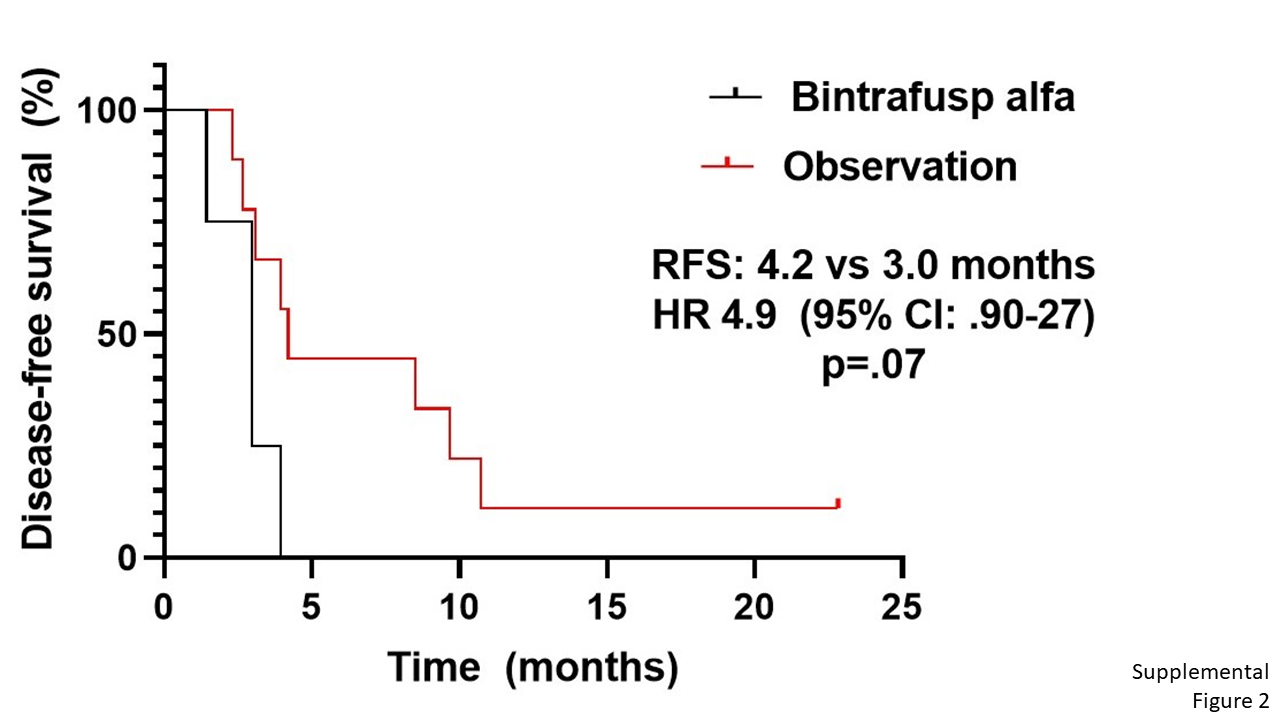

Supplement: Figure SF2 — Supplemental Figure S2 [file crc-22-0194-s06.png]
